# DNA methylation profile of inflammatory breast cancer and its impact on prognosis and outcome

**DOI:** 10.1186/s13148-024-01695-x

**Published:** 2024-07-06

**Authors:** Flavia Lima Costa Faldoni, Daniela Bizinelli, Cristiano Pádua Souza, Iara Viana Vidigal Santana, Márcia Maria Chiquitelli Marques, Claudia Aparecida Rainho, Fabio Albuquerque Marchi, Silvia Regina Rogatto

**Affiliations:** 1 Department of Clinical Genetics, University Hospital of Southern Denmark, Beriderbakken 4, 7100 Vejle, Denmark; 2 Department of Gynecology and Obstetrics, Medical School, São Paulo State University (UNESP), Botucatu, SP 18618-687 Brazil; 3 Interunit Graduate Program in Bioinformatics, Institute of Mathematics and Statistics, University of São Paulo, São Paulo, SP 05508-090 Brazil; 4 Barretos Cancer Hospital, Barretos, SP 14784-400 Brazil; 5 Department of Chemical and Biological Sciences, Institute of Biosciences, São Paulo State University (UNESP), Botucatu, SP 18618-689 Brazil; 6 Department of Head and Neck Surgery, University of São Paulo Medical School, São Paulo, SP 05402-000 Brazil; 7 Center for Translational Research in Oncology, Cancer Institute of the State of São Paulo (ICESP), São Paulo, SP 01246-000 Brazil; 8 Institute of Regional Health Research, University of Southern Denmark, 5000 Odense, Denmark

**Keywords:** DNA methylation, epigenetic regulation, Differentially methylated sites, Biomarker, Gene expression, Driver mutations

## Abstract

**Background:**

Inflammatory breast cancer (IBC) is a rare disease characterized by rapid progression, early metastasis, and a high mortality rate.

**Methods:**

Genome-wide DNA methylation analysis (EPIC BeadChip platform, Illumina) and somatic gene variants (105 cancer-related genes) were performed in 24 IBCs selected from a cohort of 140 cases.

**Results:**

We identified 46,908 DMPs (differentially methylated positions) (66% hypomethylated); CpG islands were predominantly hypermethylated (39.9%). Unsupervised clustering analysis revealed three clusters of DMPs characterized by an enrichment of specific gene mutations and hormone receptor status. The comparison among DNA methylation findings and external datasets (TCGA-BRCA stages III-IV) resulted in 385 shared DMPs mapped in 333 genes (264 hypermethylated). 151 DMPs were associated with 110 genes previously detected as differentially expressed in IBC (GSE45581), and 68 DMPs were negatively correlated with gene expression. We also identified 4369 DMRs (differentially methylated regions) mapped on known genes (2392 hypomethylated). *BCAT1, CXCL12,* and *TBX15* loci were selected and evaluated by bisulfite pyrosequencing in 31 IBC samples. *BCAT1* and *TBX15* had higher methylation levels in triple-negative compared to non-triple-negative, while *CXCL12* had lower methylation levels in triple-negative than non-triple-negative IBC cases. *TBX15* methylation level was associated with obesity.

**Conclusions:**

Our findings revealed a heterogeneous DNA methylation profile with potentially functional DMPs and DMRs. The DNA methylation data provided valuable insights for prognostic stratification and therapy selection to improve patient outcomes.

**Graphical Abstract:**

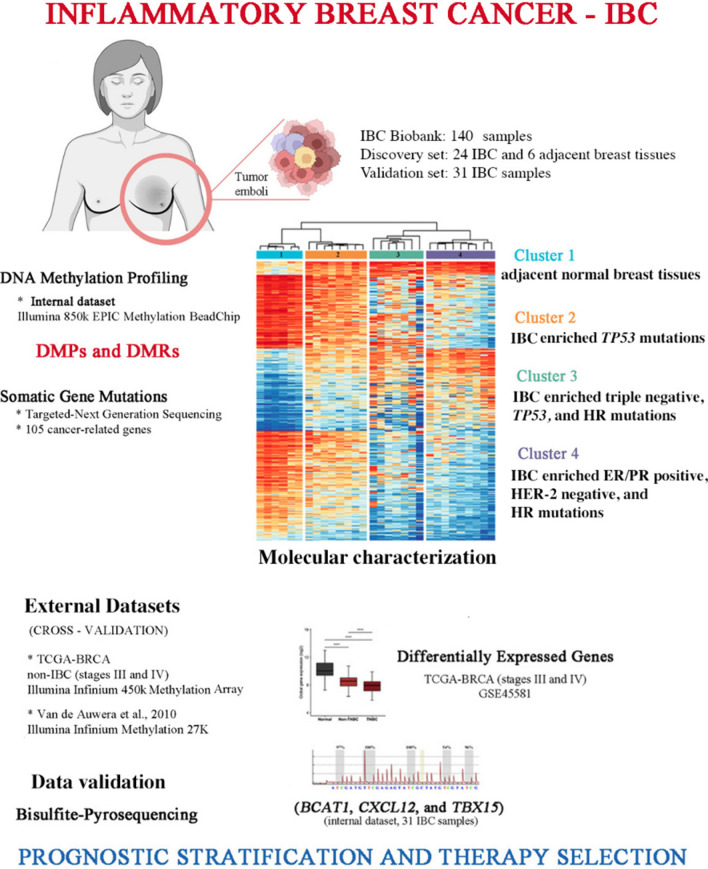

**Supplementary Information:**

The online version contains supplementary material available at 10.1186/s13148-024-01695-x.

## Introduction

Inflammatory breast cancer (IBC) is the most lethal breast cancer type (BC). IBC is characterized by rapid progression and aggressive behavior from the early stages of the disease [1]. High body mass index (BMI), insulin resistance, hormone-related factors (such as early age at menarche and pre-menopause), ethnicity (independent predictor of increased risk of breast cancer mortality), and socioeconomic status (independent predictor of advanced stages at diagnosis) have been described as risk factors associated with IBC [2, 3].

Initial efforts were undertaken to identify specific molecular signatures based on transcriptome analysis and prognostic stratification of IBC patients. Two studies showed similar gene signatures between non-IBC and IBC cases [4, 5]. Hyperactivation of interferon (alpha and gamma), hypoactivation of epidermal growth factor receptor (EGFR), TGFβ (attenuated transforming growth factor β), and p53 pathways were associated with complete pathological response [6]. Recently, Zare et al. (2021) [7] used an approach based on machine learning and reported a 50 genes signature independent of ER/HER2 status with 100% accuracy in classifying IBC cases. Frequent mutations of *TP53*, *BRCA2,* and homologous recombination (HR) genes [8, 9] and copy number alterations (gains and upregulation of *MYC* and *MDM4,* and losses of *TP53* and *RB1*) were described in IBC [9].

Although, to date, no specific molecular differences between IBC and other advanced BC types have been found, some intrinsic IBC features contribute to its aggressiveness, including stemness phenotype, high rates of motility, invasion and metastasis, tumor emboli formation leading to inflammation signals, tumor microenvironment (TME) promoting tumor epithelial cells, and immune evasion [10, 11].

DNA methylation is the most widely studied epigenetic mark. In the human genome, the enzymatic addition of the methyl radical to the carbon 5′ position of the cytosine (5-methylcytosine) occurs mainly in cytosine-phosphate-guanine (CpG) dinucleotides. DNA methylation alterations are particularly relevant in the context of global methylome changes during the development and progression of complex diseases. It is well established that weight, diet, and exercise status can influence BC risk and outcomes and that DNA methylation has a preeminent role in this process [11]. The methylation of genes involved in the inflammatory process may be associated with lifestyle and increased risk of BC development [12]. Another aspect is that DNA methylation has been associated with drug resistance, predicts response to treatment, and potentially controls the stemness phenotype [13]. Overall, these features pointed out the relevance of DNA methylation as a critical mechanism involved in IBC development and progression.

Up-to-date information is restricted to rare studies investigating DNA methylation of CpG sites associated with candidate genes showing that promoter hypermethylation is a common feature in IBC [11]. Using a 27-k microarray-based approach, Van der Auwera et al. identified two clusters characterized by low and high methylation levels; the last was enriched with metastatic and poor prognosis cases [14].

In this study, we conducted a genome-wide DNA methylation screening using a robust microarray with extensive coverage of 850,000 methylation sites across the genome to identify biologically significant differentially methylated positions (DMPs) and regions (DMRs) in IBC and their association with clinical and molecular features.

## Materials and methods

### Patients

A total of 140 IBC patients were admitted at Barretos Cancer Hospital, São Paulo, Brazil, between 2002 and 2019. We selected only biopsies collected from primary tumors prior to surgery and before chemo-radiotherapy treatment. The eligibility criteria include patients with no synchronous or metachronous tumors at diagnosis, biopsies available in the biobank, complete clinical and histopathological information in medical records, and revisions by the oncologist (C.P.S.) and pathologist (I.V.V.S.) specialists. Tumors with lymph vascular invasion and having no clinical hallmarks of IBC were excluded. The cases were classified according to the American Joint Committee on Cancer 8th Edition (AJCC 2018) [15].

According to the adopted criteria, 116 available biopsies were analyzed by an expert pathologist (I.V.V.S.) and macrodissected (to scrape off regions of tumors labeled on hematoxylin and eosin-stained tissue sections). Thirty-two cases resulted in DNA of high quality and sufficient quantity for the experiments. The study was conducted following the ethical guidelines and regulations of the Declaration of Helsinki and approved by the institutional Ethics Committee (CEP# 37779220.5.1001.5437). Written informed consent was obtained from all patients before sample collection. Clinical and epidemiological data are summarized in Table 1.

**Table 1:** Clinical and pathological characteristics of 32 female patients with inflammatory breast carcinomas evaluated in this study

Features	Number of IBC patients^b,c^ (Total = 32)
Age (years)	
≤ 50	14
> 50	18
Family history of cancer	
No	12
Yes	18
Breast and/or ovarian cancer	10
Unknown	2
Body Mass Index (kg/m^2^)	
≤ 24.9 (Normal)	6
25 to 30 (Overweight)	10
> 30 (Obese)	12
Unknown	4
Histological grade^a^	
I	1
II	19
III	12
Clinical stage	
III	19
IV	13
Hormone receptor (ER or PR)/HER2 status	
+/+	1
+/−	15
−/+	3
TNBC	13
Distant metastasis	
At diagnosis	13
During follow-up	11
Undetermined	2
Survival status	
Alive	6
Dead by disease	24
Dead by other causes	2

+: positive; −: negative; ER: estrogen receptor; PR: progesterone receptor; TNBC: triple-negative breast cancer

^a^According to Scarf Bloom Richardson grading system

^b^DNA methylation and t-NGS were performed in 24 and 29 cases, respectively

^c^Bisulfite pyrosequencing was performed in 31 cases

### DNA isolation

Genomic DNA was isolated from 32 fresh frozen macrodissected IBC tissue biopsies and six surrounding normal breast frozen tissues using the QIAsymphony kit (Qiagen, Valencia, CA, USA) at Barretos Cancer Hospital Biobank. The selected tumor area had at least 80% of tumor cells. The DNA was quantified using Qubit® dsDNA BR Assay (Life Technologies, USA).

### Targeted Next-Generation Sequencing (t-NGS)

A targeted NGS panel of 105 cancer-related genes (all exons, 3′ UTR, and 5′UTR) was performed, as previously described [10]. Twenty-one IBC samples had their mutational profile described in our previous study [9], and eight additional cases were presented herein. The libraries were prepared using SureSelectQXT Library Prep Kit (Agilent) and sequenced on the NextSeq 550 system (Illumina, San Diego, CA, USA) according to the manufacturer’s instructions. The pipeline used to select the variants was previously described [16]. In summary, we selected variants classified as pathogenic or likely pathogenic in ACMG (American College of Medical Genetics and Genomics), ClinVar (https://www.ncbi.nlm.nih.gov/clinvar/), or Clinvitae (https://www.invitae.com/en/about), and variants of uncertain significance (VUS) in ACMG presenting loss of function (e.g., frameshift, stop gain, canonical splice site). Variants classified as synonymous, intergenic or intronic regions, or mapped in the homopolymer regions were excluded. The variants were manually curated using the Genome Browse software (Golden Helix, Inc., Bozeman, MT, USA) and visualized using the ComplexHeatmap package (v.2.10.0) [17].

### DNA methylation profiling

Bisulfite conversion was performed with the EZ DNA Methylation-Gold™ Kit (Zymo Research, USA), according to the manufacturer’s recommendations. The genome-wide DNA methylation profiling of 24 IBC and six normal breast samples was evaluated using the Infinium Human Methylation EPIC BeadChip platform (Illumina, USA), according to the manufacturer’s recommendations. The Illumina HiScan system was used to scan the beadchip platform, and the data processing was performed with R software (v.4.1.0). Briefly, probes with low quality (detection *p* > 0.05), mapped in X/Y chromosomes or to SNPs, and cross-reactive were filtered out using the minfi package v.1.42.0 [18]. Differences in Type-I and Type-II probes were adjusted, and β values were normalized using the Beta MIxture Quantile dilation (BMIQ) method [19]. The batch effect was corrected with the ComBat function from the sva package (v.3.44.0) [20]. Annotation was performed with Illumina’s manifest file.

### Screening of differentially methylated sites (DMPs) and regions (DMRs)

Differentially methylated probes (DMP) comparing tumor and normal samples were identified using the limma package (v.3.50.3) [21] considering FDR-adjusted *p* < 0.05 and |∆β|≥ 0.2 annotated with the Illumina manifest file. An unsupervised K-means clustering algorithm was performed considering DMPs, while the ComplexHeatmap package was used to plot the results [17]. Differentially methylated regions (DMR) analysis was carried out using the DMRcate package (v.2.10.0) [22]. Genomic regions with at least two consecutive DMPs (distance less than 1000 nucleotides to the next CpG site) and smoothed FDR-adjusted *p* < 0.05 were considered statistically different.

### Site-specific DNA methylation evaluated by bisulfite pyrosequencing (BS-P)

Thirty-one IBC specimens (including 23 evaluated by DNA methylation array above described) and six normal breast tissues were investigated using BS-P to confirm the DNA methylation levels of *BCAT1* (cg04543413), *CXCL12* (cg06702993 and cg11267527), and *TBX15* (cg14565725, cg24884142, cg07892597, and cg10703826). The CpGs were selected based on the comparison performed in the experiments, gene function, and CpG position. The CpGs selected for *BCAT1* and *TBX15* genes were mapped in differentially methylated regions. *CXCL12* was selected based on the literature pointing out its role in triple-negative BC [23]. Primer sequences and PCR conditions used for each CpG are described in Table S1. Ten ng of gDNA bisulfite converted was amplified using the PyroMark PCR kit (Qiagen, Redwood, CA, USA), according to the manufacturer’s recommendations. The PCR products were sequenced on the PyroMark Q48 system (Qiagen, USA).

### Methylation data comparison with IBC and non-IBC external datasets

The DNA methylation data of our IBC cases were compared with those published by Van der Auwera et al. (2010) [14]. In this study, the authors analyzed 62 breast tissues (19 IBC and 43 non-IBC) in comparison with the corresponding mean β-value of 10 normal tissues generated by the Infinium Human Methylation 27 K, Illumina (data not available for download and re-analysis), considering *p* < 0.00001 and |∆β|≥ 0.17 to identify DMPs. Additionally, we explored the DNA methylation profiles (TCGA-BRCA dataset) of 178 advanced non-IBC (stages III and IV) and 93 adjacent normal samples evaluated using the Infinium Human Methylation 450 K platform (Illumina). The β values matrix and clinical information were downloaded with the TCGA biolinks package (v.2.25.2) [24] and analyzed with minfi (v.1.42.0) [17] and limma (v.3.50.3) packages [20], as previously described for our internal dataset. The intersection of the three DMP lists was acquired by drawing Venn diagrams using the ggplot2 package (v.3.4.0).

### Pathway enrichment analysis

The three final gene lists were obtained based on DMPs annotated in gene regions from the internal and external datasets comparison. Pathway enrichment analysis was performed based on the Kyoto Encyclopedia of Genes and Genomes (KEGG) database using the cluster Profiler package (v.4.4.4). Significantly enriched pathways were acquired considering FDR < 0.05, and the top results were plotted.

### Gene expression data acquisition

Public transcriptomic data of IBC samples were manually curated from the Gene Expression Omnibus repository (NCBI GEO, https://www.ncbi.nlm.nih.gov/geo/). GSE45581 was selected as the onliest dataset containing microdissected fresh frozen IBC samples from untreated patients and normal tissues [5]. To this extent, we retrieved raw probe intensity values of 20 IBC and five healthy tissue samples profiled using the Agilent Whole Human Genome 4 × 44 K array platform. The limma package was used to perform background correction and quantile normalization, and the expression data were annotated with the Agilent microarray manifest file [20]. Differentially expressed genes (DEG) were determined considering FDR < 0.05 and |FC|≥ 1.5. To our knowledge, GSE207248 was the unique public RNA-seq dataset available for in silico analysis. Herewith, count data of 22 IBC samples from untreated HER2 + patients of this dataset were downloaded and integrated with normal breast tissue samples (*N* = 22) obtained from The Cancer Genome Atlas (TCGA) and Genotype-Tissue Expression (GTEx) databases by randomly selecting 11 samples from each platform. For restricted validation purposes, we performed a variance stabilizing transformation (VST) following DESeq2 package workflow (v.1.34.0) [25], annotated gene expression matrix using biomaRt package (v.2.50.3) [26], and screened DEGs with cutoff values set to FDR < 0.05 and |FC|≥ 2.

The transcriptomic data of 196 advanced breast cancer (stages III and IV), 101 triple-negative breast cancer (TNBC), and 106 adjacent normal samples from TCGA-BRCA dataset were used to evaluate the differences in the gene expression levels of the selected targets (*BCAT1, CXCL12,* and *TBX15*). Clinical and RNA-seq count data (STAR method) were downloaded with the TCGAbiolinks package (v.2.25.2) [24], and DESeq2 package pipeline was performed for data normalization (v.1.34.0) [25]. Wilcoxon test was applied for data comparison considering *p* < 0.05 as significant.

### Statistical analysis

The β values of the DMPs identified in the internal dataset and the normalized values of the corresponding DEGs obtained from IBC samples (GSE45581) were used to determine the correspondence between methylation and expression pattern of altered genes. The statistical significance of the mean DNA methylation levels of normal versus IBC samples and IBC-TNBC versus IBC-non-TNBC samples were examined using two-way ANOVA (*p* ≤ 0.05). Data from BS-P experiments of distinct CpG dinucleotides were analyzed with the Mann–Whitney test (*p* ≤ 0.05). Logistic regression was used to estimate the association between DNA methylation levels and clinicopathological variables. Survival curves were generated using the Kaplan–Meier method and the log-rank test. Overall survival was defined as the period (in months) between the biopsy date and death. Statistical analysis was performed in Graph-Pad Prism v.9.5.1 and R software v.4.2.1 (https://www.r-project.org/). Prognostic factors underwent assessment through univariate and multivariate Cox regression analyses. Significant variables (univariate regression *p* < 0.5) and potential pairwise risk factors were incorporated into the multivariate regression model. All values related to these analyses were presented in the log_2_ scale.

## Results

### Clinical and pathological characteristics of IBC patients

The clinicopathological information of 32 women diagnosed with IBC is summarized in Table 1. The age at diagnosis ranged from 29 to 82 years, with 14 patients aged 50 years or younger and 18 patients showing a family history of cancer. According to the Body Mass Index (BMI), 22 patients were overweight or obese (BMI ≥ 25). Twelve patients presented histological grade III, 19 had clinical stage III at diagnosis, and 13 were triple-negative (TNBC: ER/PR/HER2 negatives). Thirteen patients presented distant metastases at diagnosis and eleven during follow-up. Twenty-four women died at a medium time of 18.3 months (3.06 to 105.2 months), six were alive, and two died by other causes.

Patients at clinical stage III were initially treated with neoadjuvant chemotherapy with four cycles of doxorubicin and cyclophosphamide, followed by paclitaxel for 12 weeks. In HER2-positive patients, trastuzumab was administered, and some hormone receptor-positive IBC patients also received tamoxifen or anastrozole. Patients alive after the neoadjuvant treatment were referred to surgery and adjuvant radiotherapy. Five patients presented a pathological complete response (pCR) after the neoadjuvant treatment. Stage IV patients were treated with palliative therapy, including fluorouracil, doxorubicin, and cyclophosphamide.

### Mutational profile

**Fig. 1:**
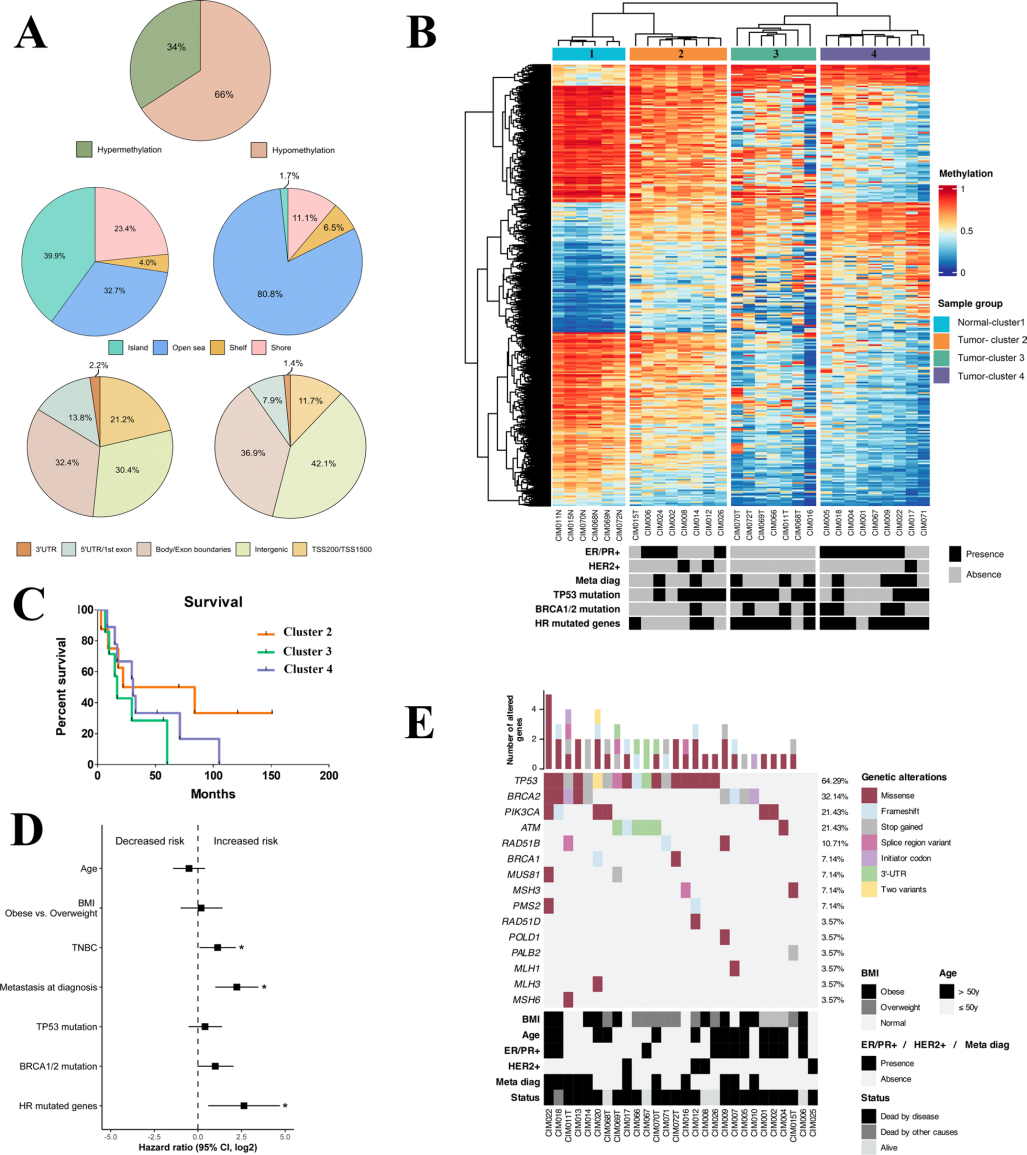
Methylation profile of the IBC internal dataset. **A** Distribution of the differentially methylated CpG probes between tumor and normal tissues (NT). The proportion of CpGs in relation to its gene location (TSS200/TSS1500, body/exons boundaries, intergenic regions, 5′UTR/first exon and 3′UTR); and to the CpG islands context (island, open sea, shelf, and shore) was based on the Illumina EPIC annotation. **B** Unsupervised K-means clustering analysis based on 46,908 identified DMPs revealed four clusters: cluster 1 is composed of adjacent normal samples and three clusters with IBC samples. Rows indicate the CpG sites, while columns represent samples. Clinical features of each case are represented below the heatmap along with targeted next-generation sequencing data for specific genes. The estrogen receptor (ER), progesterone receptor (PR), HER2 status, and the mutational pattern of *TP53, BRCA1, BRCA2,* and homologous recombination genes are indicated below the heatmap. **C** The survival curves (Kaplan–Meier and log-rank test) showed no significant statistical differences among clusters. **D** Univariate Cox regression analysis based on clinical variables and overall survival of 24 IBC patients evaluated by DNA methylation profiling. The forest plot shows the hazard ratios (squares), and the horizontal bars represent the range between the lower and upper limits of the 95% confidence intervals (CI) in the log_2_ scale. IBC patients positive for estrogen (ER), progesterone (PR), and or human epidermal growth factor receptor 2 (HER2) were taken as a reference against triple-negative tumors. In the analysis of the BMI variable, we excluded patients within the normal weight range due to the small sample size (*N* = 4); overweight patients were used as a reference. For the remaining variables, we considered the absence of the corresponding predictive factor as a reference for analysis (*p < 0.05). **E** A panel of 105 cancer-related genes was investigated in 28 IBC patients using t-NGS. Highlights include clinical, molecular, and vital status information. Genes are organized in descending order of alterations. The top bar plots illustrate the number of altered genes detected in each sample, and the percentages on the right indicate the number of samples with genetic alterations for a given gene among all analyzed samples. Genes without selected variants were excluded

The mutational profile was performed in 28 IBC samples (21 previously reported) [9] using t-NGS (105 cancer driver genes). The most common variants were detected in *TP53* (18 cases), *BRCA2* (9 cases), and *PIK3CA* (6 cases) (Fig. 1E). Interestingly, 20 out of 28 cases showed variants in genes involved in the homologous recombination pathway (Table S2).

### Identification of DMPs and DMRs in IBC

The DNA methylation performed in 24 IBC and six adjacent normal breast samples (NT) revealed 46,908 DMPs (FDR < 0.05 and |∆β|≥ 0.2), in which 30,919 (66%) were hypomethylated and 15,989 (34%) hypermethylated (Fig. 1A). Hypermethylation was predominant in the CpG islands (39.9%), followed by open sea regions (32.7%), while most hypomethylation was found in the open sea region (80.8%). Hypermethylated sites (32.4%) were frequently found in CpGs mapped in gene bodies and hypomethylated DMPs in intergenic regions (42.1%). Hypermethylated CpGs mapped in promoter regions (21.2%) were more frequently observed than in hypomethylated DMPs (11.7%). Annotations related to shore, shelf, and other regions are illustrated in Fig. 1A. Supplementary Fig. 1 summarizes the study design and main findings.

The unsupervised clustering analysis of all DMPs revealed four clusters: One grouped all normal samples (cluster 1), and three had IBC samples (Fig. 1B, Table S3). Clinical and molecular features distribution, such as estrogen (ER) and progesterone receptors (PR), HER2 status, metastasis at diagnosis, *TP53, BRCA1*, *BRCA2*, and HR genes mutations were distributed among the three clusters (Fig. 1B). Cluster 3 was enriched with triple-negative cases, *TP53* mutation, and cluster 4 with ER/PR positive, HER2-negative. Both clusters 3 and 4 were enriched with HR mutated genes. Although no significant difference was observed in the survival curves and BMI among these groups of patients, cluster 3 presented shorter survival (Fig. 1C; Table S4).

The Cox regression model was applied to evaluate the prognostic role of molecular and clinical information gathered from our cohort of IBC cases screened for DNA methylation. We found that TNBC (*p* = 0.036; HRatio = 1.131; CI 0.095–2.175), metastasis at diagnosis (*p* = 0.00048; HRatio = 2.23; CI 0.993–3.466), and HR mutated genes (*p* = 0.011; HRatio = 2.64; CI 0.588–4.700) were significantly correlated with overall survival as independent factors for increased risk (Fig. 1D). Multivariate regression analysis revealed that only metastasis at diagnosis (*p* = 0.0032; HRatio = 2.10; CI 0.704–3.487) remained a prognostic indicator for overall survival. A pairwise comparison of potential variables showed a robust association between triple-negative tumors (*p* = 0.015; HRatio = 1.45; CI 0.288–2.622) and metastasis at diagnosis (*p* = 0.0003; HRatio = 2.57; CI 1.178–3.969), indicating a poorer overall survival. Similar findings were observed in patients presenting metastasis at diagnosis (*p* = 0.0073; HRatio = 1.71; CI 0.460–2.953) and HR genes mutation (*p* = 0.0305; HRatio = 2.36; CI 0.222–4.499). Cox regression analysis highlighted the significant role of metastasis at diagnosis in predicting poorer overall survival. Furthermore, our model underscores that the separate interaction of this variable with TNBC and tumors with HR mutations is significantly associated with an increased risk of death.

**Fig. 2:**
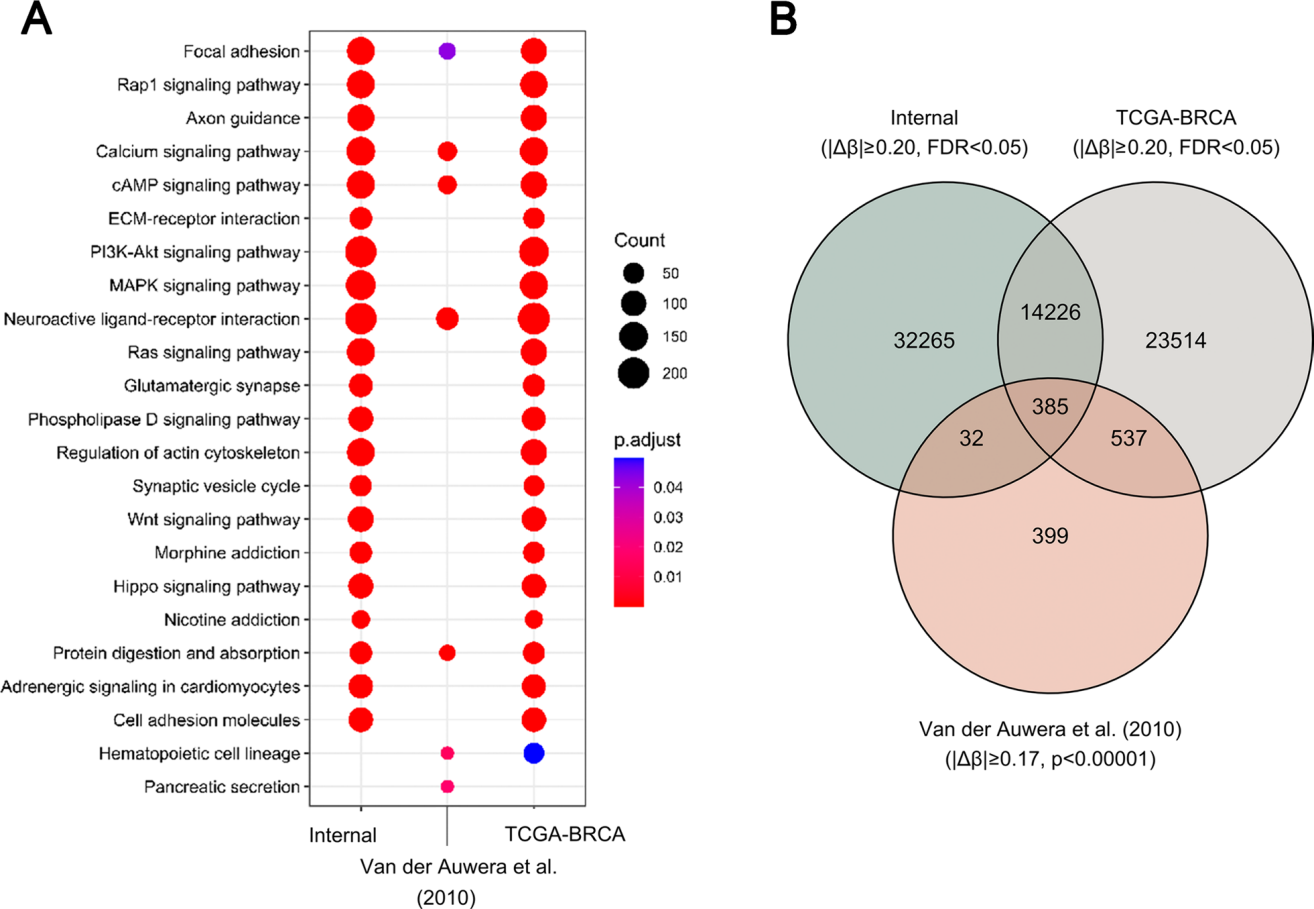
Differentially methylated probes (DMPs) comparison performed among the internal dataset, 27 k Van der Auwera et al. (2010), and TCGA-BRCA advanced tumors. **A** KEGG pathway analysis shows five shared enriched pathways among three datasets. **B** Venn diagram obtained from dataset comparisons (internal dataset, TCGA-BRCA, and Van der Auwera et al., 2010) shows 385 shared DMPs among the datasets comparison

Among the 46,908 significant DMPs, 17,868 CpGs were mapped in intergenic regions and 29,040 (11,135 hypomethylated and 17,905 hypermethylated) in known genes. The DNA methylation data is available in the Gene Expression Omnibus database (GSE238092). The enrichment analyses showed pathways involved in signal transduction (MAPK signaling pathway, PI3K-Akt signaling pathway, Ras signaling pathway), cellular adhesion and movement (focal adhesion, ECM-receptor interaction, regulation of actin cytoskeleton, cell adhesion molecules), and neuronal system (axon guidance, neuroactive ligand-receptor interaction, glutamatergic synapse, synapse vesicle cycle, morphine addiction, and nicotine addiction), among others. The top 20 KEGG pathways are shown in Fig. 2A.

A total of 4369 DMRs mapped on known genes was found (2392 DMR hypomethylated and 1977 hypermethylated) (Table S4). Two genes presented more than 30 CpGs in the same DMR, *TBX15* (36 CpGs) and *OR2I1P* (31 CpGs).

### Comparison of DNA methylation profiles of IBC and non-IBC using external datasets

The comparison of our findings with the Van der Auwera et al. (2010) [27] data (1353 DMPs; *p* < 0.00001, |∆β |≥ 0.17) revealed 417 shared DMPs with the same methylation status, 92 hypomethylated and 325 hypermethylated (Figure S1). Using the same criteria adopted in the internal dataset (FDR < 0.05 and |∆β|≥ 0.2) to analyze advanced breast tumors from TCGA, we found 38,662 DMP, in which 14,611 DMP were found in both datasets (6298 hypomethylated and 8313 hypermethylated). The comparison among these three datasets resulted in 385 shared DMPs (Fig. 2B) mapped in 333 genes, 69 hypomethylated and 264 hypermethylated (Table S5). Enrichment analysis revealed five main pathways: Focal Adhesion, Calcium Signaling Pathway, cAMP Signaling Pathway, Neuroactive Ligand-Receptor Interaction, and Protein Digestion and Absorption (Fig. 2A, Table S6).

The gene expression data of IBC samples retrieved from the GSE45581 (Whole Human Genome Microarray 4 × 44 K Agilent platform) was compared with our DNA methylation results (Pearson correlation, *p* < 0.05), revealing 151 DMPs mapped in 110 genes; 68 probes mapped in 50 genes presented a negative correlation (Table 2). The gene annotation using CancerMine (http://bionlp.bcgsc.ca/cancermine/) and MSigDB (https://www.gsea-msigdb.org/gsea/msigdb/) databases showed 50 genes, of which 31 are cancer-related (Table 2). We also accessed the expression levels of these genes using the GSE207248. Ten genes were hypomethylated and overexpressed (such as the oncogenes *CHST11, KIF26B, LAIR1, NTM, RUNX2, VOPP1,* and *WDR5*), and six hypermethylated and down expressed (including the tumor suppressor genes *CDO1, CTDSPL, EBF1, ROBO3,* and *TGFBR3)* (Table 2).

**Table 2:** List of probes and genes with a negative correlation between the internal dataset and expression data from the GSE45584. Significant values of correlation are presented (*p* < 0.05)

Probe	Gene	Δβ (T-N)^a^	Correlation	Expression	CancerMine^c^	MSigDB Hallmarks^d^
				(T-N) FC^b^		
				GSE207248		
cg09302836	*ALPK2*	−0.23	−0.4994	4.83		
cg13347825	*ARID5B*	−0.30	−0.4487	−2.65	Oncogene	Androgen response
cg22863838		−0.26	−0.5904			
cg20270052		−0.24	−0.7361			
cg06066117	*ARSB*	−0.31	−0.5871	2.35		
cg01376622	*CD163*	0.24	−0.5378	–	Oncogene	
cg27275193	*CDNF*	−0.29	−0.5339	−3.41		
cg16265906	*CDO1*	0.31	−0.4715	−32.94	TSG	Xenobiotic metabolism
cg11036833		0.33	−0.4871			
cg02792792		0.33	−0.4809			
cg21377673		0.34	−0.4795			
cg07644368		0.4	−0.4911			
cg11195884	*CHST11*	−0.27	−0.5405	2.01	Oncogene	
cg07271809		−0.24	−0.4609			
cg07087674	*CLIP4*	−0.26	−0.5443	−4.70		
cg17572907	*CRIM1*	−0.24	−0.4962	−5.93	TSG	
cg05090717	*CTDSPL*	0.21	−0.5935	−2.86	TSG	
cg08171483		0.22	−0.5001			
cg07993092	*CTTNBP2*	−0.23	−0.5005	−3.86		
cg26596307	*CX3CL1*	−0.26	−0.5691	−6.97	Driver	Apical junction
cg02485328	*EBF1*	0.22	−0.4904	−5.31	TSG	
cg25153092	*FAM49B*	0.24	−0.488	–		
cg19238325	*GPR62*	−0.28	−0.5262	–		
cg03150042	*ITGB2*	0.24	−0.6187	–	Oncogene	Mtorc1 signaling
cg15146125	*ITPRIPL1*	0.26	−0.4912	–		
cg08983217		0.32	−0.5335			
cg06909469		0.32	−0.4817			
cg17932631		0.35	−0.5566			
cg24161793		0.38	−0.5018			
cg05497345		0.38	−0.5049			
cg12098156	*CEMIP/KIAA1199*	−0.25	−0.5505	–		
cg00643651	*KIF26B*	−0.24	−0.5323	23.93	Oncogene	
cg12562259	*LAIR1*	−0.33	−0.5311	2.20	Oncogene	
cg11063877	*LARP4*	−0.34	−0.5504	–	TSG	
cg01928411		−0.31	−0.5669			
cg25440344	*LIFR*	−0.32	−0.4690	−15.72	TSG	Adipogenesis
cg06756172		−0.22	−0.5243			
cg15460135	*LILRA3*	−0.31	−0.5938	–		
cg19802477	*LILRA4*	−0.28	−0.4701	–		
cg02793158	*LIMS2*	0.24	−0.4624	−6.86		
cg20174927	*MME*	−0.29	−0.4755	−12.07	TSG	
cg12638731	*NBEA*	0.38	−0.4691	2.49		
cg17339550	*NTM*	−0.32	−0.4489	2.70	Oncogene	EMT
cg22968327	*NUP93*	−0.28	−0.4708	–	Driver	Interferon gamma response
cg18494134	*PACSIN1*	0.35	−0.5330	2.12		
cg11466109		0.42	−0.4608			
cg07347315	*PDLIM1*	−0.3	−0.4922	2.23		Reactive oxygen species pathway
cg11046854	*PMEPA1*	−0.29	−0.5002	–	TSG	TNFα signaling via NFKB
cg22642777	*PPP2R2B*	0.39	−0.4853	–		
cg21334734	*RAB10*	−0.24	−0.4663	–	Oncogene	
cg10143911	*RAB7A*	−0.37	−0.4544	−3.37	Oncogene	
cg11730618		−0.26	−0.7315			
cg23313157	*RAI14*	−0.23	−0.5513	–	Oncogene	
cg15383276	*RASL10A*	0.21	−0.4657	–	TSG	
cg20085261	*RBM5*	0.24	−0.4704	–	TSG	Heme metabolism
cg02940147	*ROBO3*	0.27	−0.4848	−3.22	TSG	
cg01126481	*RUNX2*	−0.29	−0.4874	3.61	Oncogene	
cg10569823	*SORBS1*	−0.26	−0.4944	−9.76	Oncogene	Adipogenesis
cg17080882	*TGFBR3*	0.31	−0.5501	−16.47	TSG	Apoptosis
cg08671821	*TMEM165*	−0.24	−0.5608	–		
cg25431366	*UBE2F*	0.22	−0.7734	–	Oncogene	
cg17901038	*UBE2O*	0.21	−0.6354	–	Oncogene	
cg16330742		0.28	−0.5013			
cg11192933	*UGGT1*	−0.25	−0.5261	–		
cg17066594	*UNC93B1*	0.25	−0.4527	–		
cg00487299	*VANGL1*	−0.26	−0.4872	–	Oncogene	
cg21959598	*VOPP1*	−0.38	−0.4492	2.38	Oncogene	
cg03473042	*WDR5*	−0.24	−0.5402	2.30	Oncogene	

*TSG* Tumor suppressor gene, *EMT* Epithelial mesenchymal transition

^a^Negative value: hypomethylated probes; Positive value: hypermethylated probes

^b^FC: fold change; negative values: decreased expression; positive value: increased expression

^c^Available in http://bionlp.bcgsc.ca/cancermine/

^d^Molecular Signatures Database available https://www.gsea-msigdb.org/gsea/msigdb/

### Locus-specific DNA methylation analysis

**Fig. 3:**
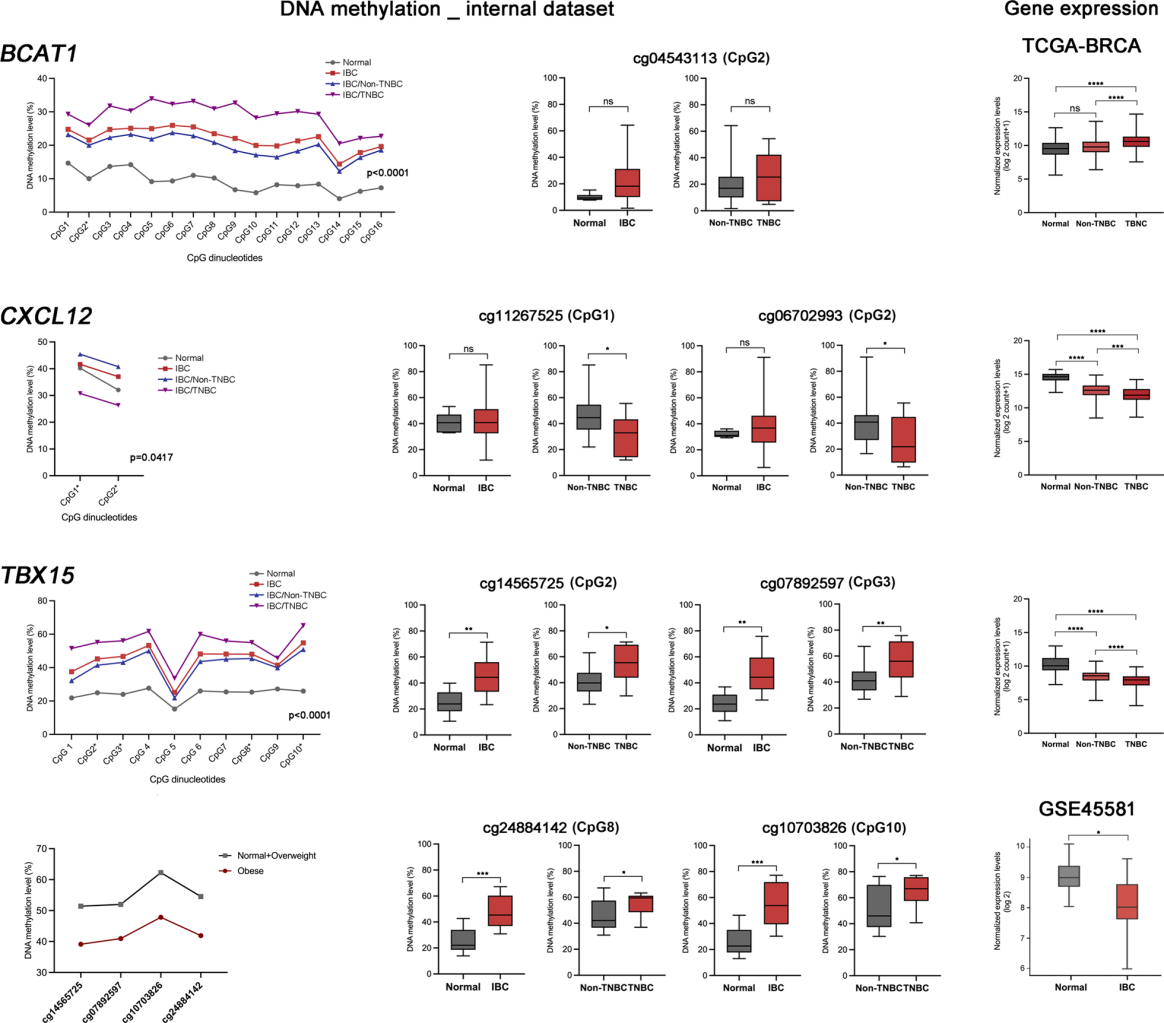
Loci-specific DMRs analyzed by bisulfite pyrosequencing of CpGs associated with selected genes (*BCAT1*, *CXCL12*, and *TBX15)* using the internal cohort and cross-validation with gene expression levels using external cohorts (advanced III-IV stages from TCGA-BRCA and IBC from GSE45581). The first column shows the comparison of the means of DNA methylation levels of each associated DMRs containing the interrogated CpG probes and the flanking CpG dinucleotides between normal and IBC samples. Tumor samples were also dichotomized in non-TNBC and TNBC, and the methylation levels were compared. The DNA methylation levels of distinct DMPs confirmed higher methylation levels of *BCAT1* and *TBX15* genes and lower levels of *CXCL12* in IBC. The methylation levels of *TBX15* gene were associated with the subtype TNBC and obesity (*p ≤ 0.05, **p ≤ 0.01; ***p ≤ 0.001, and ****p ≤ 0.0001). ns: not significant (*p* > 0.05)

Bisulfite pyrosequencing was used to quantitatively determine the methylation of CGs mapped on *BCAT1*, *CXCL12*, and *TBX15* genes. Figure 3 shows the mean DNA methylation levels of consecutive CpG dinucleotides flanking those interrogated by microarrays as well as the methylation levels of individualized DMPs. The analysis of gene body DMR encompassing 16 CpGs of *BCAT1* gene confirmed increased methylation levels in IBC compared with normal samples. We also detected a trend to increase methylation levels in IBC versus normal tissues (*p* = 0.0725), but no differences were observed between triple-negative IBC compared with non-triple-negative IBC. The comparison with the expression levels of *BCAT1* of TCGA-BRCA stages III-IV revealed no difference between non-TNBC and normal samples. However, a significant increase in expression was observed between normal *versus* TNBC and non-TNBC *versus* TNBC.

Based on the unsupervised K-means clustering analysis of DMPs and in the literature data, two differentially methylated CpGs (cg06702993 and cg11267527) of *CXCL12* were evaluated. Triple-negative IBC showed a significant decrease in DNA methylation levels of *CXCL12* compared to non-triple-negative samples (*p* = 0.0481). These findings are in accordance with the expression levels of *CXCL12* of TCGA-BRCA stages III-IV, showing a decreased expression in advanced tumors compared to normal samples and TNBC.

We selected four differentially methylated CpGs extracted from the DMR of *TBX15* (10 analyzed*)*, which were also altered in TCGA-BRCA dataset. We confirmed increased methylation levels in IBC compared to normal samples. Triple-negative tumors presented increased methylation levels of *TBX15* compared with non-triple-negative IBC cases. The expression level of *TBX15* was confirmed as significantly decreased in advanced BC tumors (TCGA-BRCA stages III-IV) and IBC (GSE45581) compared to normal samples (Fig. 3G). In addition, we found a significant negative association between methylation levels of *TBX15* and obesity (cg14565725, OR 0.9182, CI 0.8332 to 0.9864, *p* = 0.0004; cg07892597, OR 0.9391, CI 0.8661 to 0.9996, *p* = 0.0486; cg10703826 OR 0.9336, CI0.8690 to 0.9892, *p* = 0.0184; and cg24884142, OR 0.8864, CI 0.7909 to 0.9659, *p* = 0.0042).

## Discussion

Cancer-specific alterations in DNA methylation are closely related to a variety of malignancies. Although the main risk factors associated with IBC (such as obesity, exposition to endocrine disruptors, socioeconomic status, and racial disparities) have been linked with epigenetic alterations, limited DNA methylation data are available in the literature [11].

Using a robust microarray platform with extensive coverage of CpG islands, genes, and enhancers (850 k Infinium Methylation EPIC BeadChip platform, Illumina), we verified that 66% of the DMP were hypomethylated mainly in open sea regions, while 34% were hypermethylated frequently in CpG islands. CpG islands hypermethylation was previously reported in IBC [14]. Genome-wide DNA hypomethylation have been described in cancer cells and associated with chromosomal instability, de-repression of imprinted genes and retrotransposons, and aberrant gene expression [27]. The high proportion of hypomethylated CpG sites in the gene body is a feature of many tumors, but its biological effect is still under investigation. Gene body methylation suppresses gene expression through chromatin and its interaction with regulatory elements (alternative promoters, enhancers, transcription factors, and repetitive elements) [28]. Gene body methylation can also activate transcription by suppressing spurious gene transcription, regulating, and allowing the correct transcription process [29, 30]. Hypermethylation in gene bodies has been associated with increased oncogenes expression and related to tumor progression [30].

Unsupervised hierarchical clustering analysis of DNA methylation showed a perfect separation between normal and IBC samples. Three clusters composed exclusively of IBC samples were found, and their clinical features were compared. Body mass index (BMI) is recognized as an independent risk factor for IBC development. However, no significant difference was observed among IBC clusters, probably due to the small sample size. Although no difference was observed in the overall survival among IBC clusters, cluster 3 (TNBC enriched) showed a trend toward shorter survival than clusters 2 and 4. DNA methylation profiles in IBC could provide new molecular stratification with potential implications for individual patient prognosis and therapy selection.

Cluster 3 was enriched by TNBC subtype (7 of 9 cases), *TP53* mutations, and homologous recombination mutated genes. Triple-negative BC is more frequently detected in IBC than the other subtypes, and it is associated with worse overall survival, distant metastasis, and locoregional relapse [31]. Gene expression datasets available in GEO showed no differences in the comparison between triple-negative IBC and triple-negative non-IBC, which suggested a molecular similarity [32]. A recent report showed that TNBC subtype luminal androgen receptor subtype presented the most hypermethylated CpGs, while the mesenchymal subtype the most hypomethylated CpGs [33]. Differences among the triple-negative IBC subtypes should also be explored and contribute to developing new treatment strategies for this devastating disease.

The relationship between clinical variables and outcomes of our IBC cohort (univariate and multivariate Cox proportional-hazards regression models) revealed that the TNBC subtype, metastasis at diagnosis, HR mutations were significantly correlated with overall survival. However, only metastasis at diagnosis remained a significant prognostic factor in the multivariate analysis. Like our findings, Dano et al. (2021) utilized multivariate analysis and demonstrated that overall survival was significantly shorter in metastatic IBC cases compared with metastatic non-IBC [34]. Deficiency in homologous recombination pathway, such as *BRCA2* mutations frequently altered in our cases, has been described in IBC cases [8, 9]. The limited sample size may have contributed to the lack of statistical significance of our parameters since the TNBC phenotype is a well-known risk factor for unfavorable prognosis in breast cancer and IBC [35, 36].

Fifteen IBC patients presented somatic *TP53* mutations distributed into the three clusters. *TP53* and HR mutations are frequently associated with triple-negative BC [37], a finding also detected in our IBC samples. The impact of somatic *TP53* mutations on the prognosis was reported as independent of hormone receptor status [38], a feature also observed in our dataset. Somatic *TP53* mutations alter the level of transcripts like thymine-DNA glycosylase that are implicated in the physiological control of promoter demethylation of several genes involved in embryogenesis and development [39]. Promisor cancer treatment strategies have been described based on targeting dysfunctional p53, reactivating the mutant to its wild-type form, and inhibiting the interaction between wild-type p53 and MDM2/MDM4 [40]. Antagonists of p53-MDM2/MDM4 are undergoing clinical trials. Moreover, two mutant p53-reactivating compounds have progressed to clinical trials, COTI-2 and APR-246 [40]. These strategies could be tested in IBC in conjunction with other therapies to improve outcomes.

We found an enrichment of homologous recombination genes mutation in clusters 3 (6 of 7 IBC cases) and 4 (8 out of 9 IBC cases). Although not statistically significant, both showed shorter survival compared to cluster 2. Homologous recombination is the most effective mechanism to repair double-stranded DNA breaks (DSB). Mutations leading to homologous recombination deficiency increase the degree of genetic instability and the emergence of other mutations that potentially promote tumor progression. Using human and mouse cells and DNA damage induced by ionizing radiation, Moureau et al. (2016) provide evidence that p53 is associated with the regulation of DSB repair [41]. Patients with somatic and germline *BRCA1/2* mutations, the leading players of the HR pathway, have sensitivity to platinum-based chemotherapy and PARP inhibitors, an alternative to treat IBC patients. IBC models demonstrated that PARP inhibitors improved radiotherapy's effectiveness and provided the preclinical rationale for the phase II randomized trial for IBC patients [42]. The molecular classification of a large cohort of IBC cases according to mutations and DNA methylation data may help identify biomarkers and design therapeutic strategies to improve patient outcomes. Several epigenetic drugs are under investigation in clinical trials in different tumor types and could be an efficient alternative to treat IBC patients.

We also identified 4369 DMRs mapped on known genes (2392 DMR hypomethylated and 1977 hypermethylated). Two genes showing DMRs were selected for data confirmation using BS-P: *BCAT1* and *TBX15.* Increased methylation levels of 16 CpGs (gene body) of *BCAT1* in IBC were compared with normal samples. *BCAT1* expression levels of TCGA-BRCA stages III-IV samples were significantly increased comparing normal *versus* TNBC and non-TNBC *versus* TNBC. *BCAT1* levels have been positively associated with tumor progression and worse prognosis in invasive breast cancer and TNBC [43]. In a pan-cancer study with 16,847 samples, Li et al. (2022) [44] explored the expression, potential mechanisms, and clinical significance of *BCAT1*. *BCAT1* was predominantly expressed in ERα-negative/HER-2-positive breast cancer and TNBC, and its overexpression enhanced the capacity of antiestrogen-sensitive cells to grow in the presence of antiestrogens [44]. The silencing of *BCAT1* in an orthotopic TNBC xenograft model resulted in a massive tumor volume reduction in vivo [44]. Increased *BCAT1* expression has been associated with unfavorable prognosis in several tumor types, including BC, and it has been suggested its potential as an immunotherapeutic cancer marker.

Ten CpGs of the DMR mapped on the *TBX15* (T-box transcription factor 15) gene were also confirmed as hypermethylated in IBC compared with normal samples. Also, TN IBC samples presented increased hypermethylation compared with non-IBC and normal samples. *TBX15* encodes a transcript factor associated with mesodermal differentiation, skeleton development, and adipocyte differentiation. Aberrant *TBX15* methylation has been described in various tumors, such as prostate [45], ovarian [46], and hepatocellular carcinomas [47]. Recently, a study analyzed the functionality of DNA methylation in a DMR region of *TBX15* immediately upstream and downstream of the unmethylated promoter region in myoblasts and skeletal muscle [48]. The authors showed that hypermethylated DMR suppresses enhancer/extended promoter activity and down modulates gene expression without silencing it [48]. Importantly, the *TBX15* DMR overlaps with cis-regulatory elements, and the DMPs validated by BS-P are located close to the eight best candidates’ functional single nucleotide polymorphisms (SNPs) for obesity-trait or osteoporosis risk [49]. Significantly lower *TBX15* expression level was observed in TNBC (TCGA-BRCA) than in non-TNBC and normal tissues. Moreover, IBC showed significantly decreased expression levels compared with normal samples (GSE45581). We showed a significant association between *TBX15* methylation levels and obesity. BMI is an independent and positively associated risk factor for IBC [3]. *TBX15* is a novel adipose master transcription factor [50]. Its knockdown in human primary preadipocytes resulted in changes in the expression of 130 network genes, including the key adipose transcription factors. We suggest that *TBX15* is a key gene regulated by DNA methylation that potentially contributes to obesity in IBC patients.

Bisulfite pyrosequencing was also used to evaluate two CpGs mapped on *CXCL12. CXCL12* belongs to the chemokine family, and its expression level has been associated with poor prognosis in TNBC [50]. We observed that TN IBC showed lower DNA methylation levels than non-TN IBC and normal samples. In BC at advanced stages from TCGA-BRCA dataset, *CXCL12* expression levels were higher in TNBC than in non-TNBC. Increased levels of *CXCL12* promote the accumulation and amplification of myeloid-derived suppressor cells, M2-phenotype macrophages, and regulatory T cells, which contributes to the induction of an immunosuppressive/anergic tumor-permissive environment to benefit tumor formation in TNBC [50]. The authors targeted the delivery of antagonists and the disruption of *CXCL12/CXCR4* signaling in the tumor tissue, which promoted depletion of the stromal barrier and relieved immunosuppression, and the T cell infiltration into the tumor site, enhancing the efficacy of immune checkpoint blockade therapy in TNBCs [50].

The enrichment analysis of the internal DMP showed pathways previously reported in IBC biology, such as focal adhesion, extracellular matrix interaction, regulation of actin cytoskeleton, and cell adhesion molecules. These pathways are related to cell–cell contact between cancer cells or cancer cells and tumor microenvironment cells required for forming the IBC emboli structure and the tumor cell clusters with high metastatic potential [10]. The PI3K-AKT signaling pathway is involved in breast cancer progression and associated with cell motility and actin reorganization in IBC [51]. Alterations in MAPK pathway genes were associated with IBC aggressiveness, increased cell proliferation, stemness, and metastatic potential [51]. RAS signaling pathway initiates cell motility and focal adhesions in aggressive BC [52]. These data suggest that IBC aggressiveness features may be controlled by methylation mechanisms in critical genes closely related to cancer progression and worse prognosis.

We also found pathways related to nervous system development and neuronal signaling encompassing nine differentially methylated genes (*CDNF, NBEA, NTM, PACSIN1, PPP2R2B, RAB7A, RASL10A, ROBO3,* and *VANGL1*). The nerve-cancer crosstalk suggests an association with intratumoral neural infiltration. The nervous system modulates angiogenesis, the tumor microenvironment, bone marrow, immune functions, and inflammatory pathways to influence metastases [53]. These neural signaling altered by DNA methylation in IBC may relate to tumor progression and metastasis.

## Conclusions

We found a distinct DNA methylation profile in triple-negative inflammatory breast tumors. The methylation pattern of *CXCL12*, *BCAT1,* and *TBX15* genes was confirmed by bisulfite pyrosequencing. We suggest that *TBX15* is a potential marker regulated by DNA methylation and related to obesity in IBC. Also, nervous system development and neuronal signaling pathways were involved in IBC and should be further investigated to amplify the therapeutic options of this aggressive and morbid disease.

### Supplementary Information

**Figure S1** Workflow representative of the strategy used in our DNA methylation analysis in inflammatory breast cancer (IBC). (A) DNA methylation comparing our data (internal dataset), breast cancer stages III and IV from TCGA, and Van der Auwera et al. (2010) study revealed 385 shared differentially methylated probes (DMPs). Gene expression of IBC evaluated with the Agilent microarray platform (GSE45581, available in Gene Expression Omnibus) revealed 2480 differentially expressed genes (DEGs). Integrative analysis between DMPs of the internal dataset and DEGs (GSE45581) with significant Pearson correlation resulted in 151 DMPs. (B) Strategy used to select candidates investigated by bisulfite pyrosequencing. Four selected CpGs differentially methylated of the *TBX15* gene were altered in the TCGA-BRCA dataset; three CpGs of the *CXCL12* gene were differentially methylated in triple-negative cases compared with non-triple-negative IBC cases, and *BCAT1* presented four CpGs differentially methylated and was differentially expressed (integrative analysis)

**Table S1** Primer sets used in the bisulfite pyrosequencing reaction for CpGs mapped in the differentially methylated regions of *TBX15, CXCL12,* and *BCAT1.*

**Table S2** Genetic variants found in *TP53*, *PIK3CA*, homologous recombination and mismatch repair genes among the 29 IBC cases assessed by t-NGS.

**Table S3** Differentially methylated regions identified in the analysis of 24 inflammatory breast carcinomas.

**Table S4** Body mass index (Kg/m2) and overall survival among tumor clusters.

**Table S5** Shared differentially methylated probes and delta-Beta values among our internal dataset, 27 k study (Van der Auwera et al., 2010) [14] and Advanced Breast Cancer (TCGA-BRCA dataset). Significant threshold with *p* values < 0.05.

**Table S6** KEGG pathway enrichment analysis revealed five pathways shared among DMPs of our internal dataset, 27 K Study (Van der Auwera et al., 2010), and TCGA-BRCA.

## Data Availability

Data are reported in detail. Further data are available upon academical request.

## Abbreviations

ACMG: American College of Medical Genetics and Genomics
BMI: Body mass index
BMI: Beta MIxture Quantile dilation
CI: Confidence interval
CpG: Cytosine-phosphate-guanine
DEG: Differentially expressed genes
DMP: Differentially methylated probes
DMR: Differentially methylated regions
DSB: Double-stranded DNA breaks
EGFR: Epidermal growth factor receptor
ER: Estrogen receptor
GEO: Gene Expression Omnibus
GTEx: Genotype-Tissue Expression
HR: Homologous recombination
HRatio: Hazard Ratio
IBC: Inflammatory breast cancer
KEGG: Kyoto Encyclopedia of Genes and Genomes
LP: Likely pathogenic variant
OR: Odds Ratio
P: Pathogenic variant
pCR: Pathological complete response
TCGA: The Cancer Genome Atlas
TGFβ: Attenuated transforming growth factor β
TME: Tumor microenvironment
TNBC: Triple-negative breast cancer
t-NGS: Targeted next-generation sequencing
VST: Variance stabilizing transformation
VUS: Variants with uncertain significance
