# Supplementary figures and images for: DNA methylation profile of inflammatory breast cancer and its impact on prognosis and outcome
Source: Clin Epigenetics. 2024 Jul 6;16:89. doi: 10.1186/s13148-024-01695-x (PMC11227707; doi:10.1186/s13148-024-01695-x)

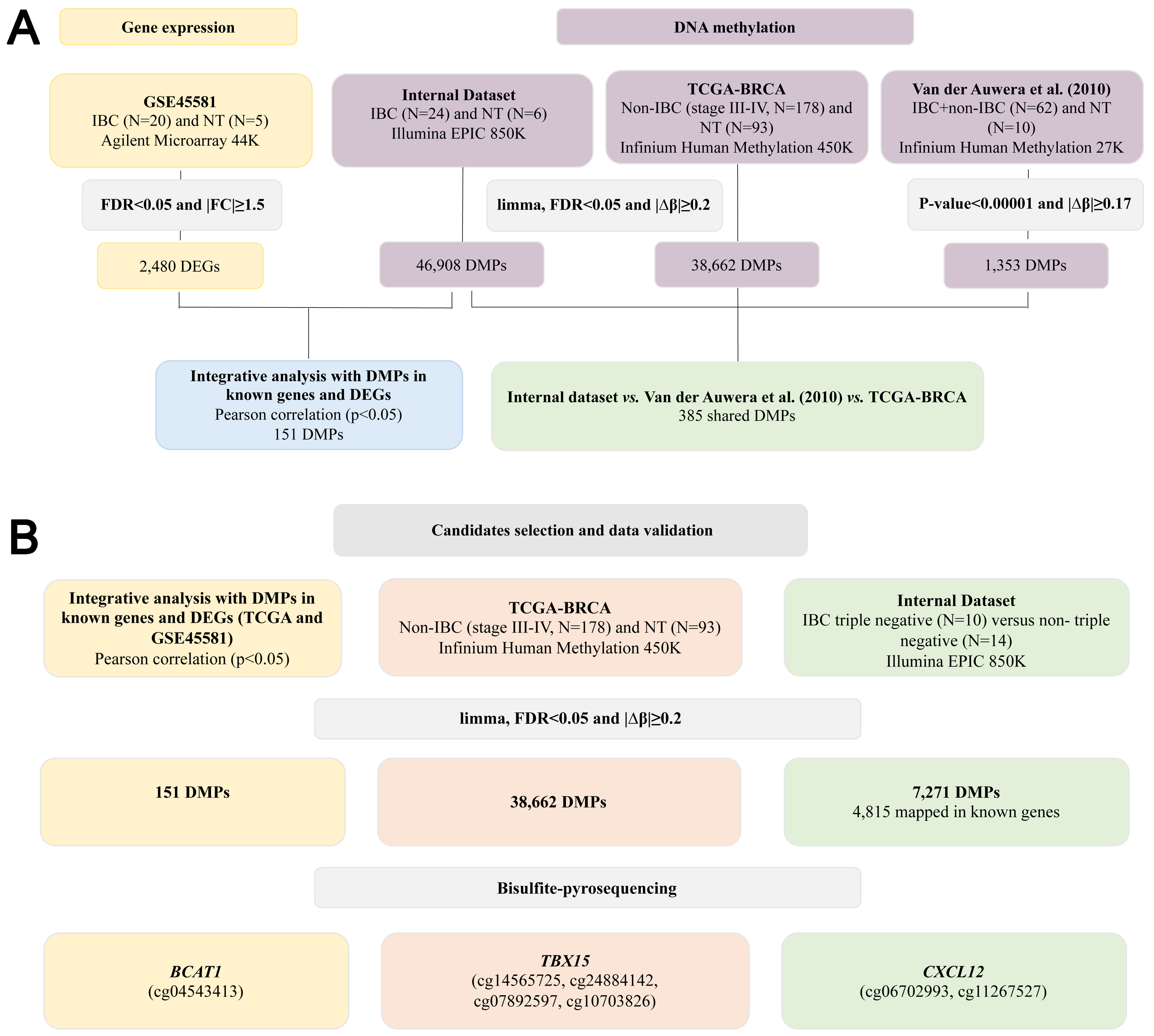

Supplement: Supplementary file 1 — Figure S1 Workflow representative of the strategy used in our DNA methylation analysis in inflammatory breast cancer (IBC). (A) DNA methylation comparing our data (internal dataset), breast cancer stages III and IV from TCGA, and Van der Auwera et al. (2010) study revealed 385 shared differentially methylated probes (DMPs). Gene expression of IBC evaluated with the Agilent microarray platform (GSE45581, available in Gene Expression Omnibus) revealed 2480 differentially expressed genes (DEGs). Integrative analysis between DMPs of the internal dataset and DEGs (GSE45581) with significant Pearson correlation resulted in 151 DMPs. (B) Strategy used to select candidates investigated by bisulfite pyrosequencing. Four selected CpGs differentially methylated of the TBX15 gene were altered in the TCGA-BRCA dataset; three CpGs of the CXCL12 gene were differentially methylated in triple-negative cases compared with non-triple-negative IBC cases, and BCAT1 presented four CpGs differentially methylated and was differentially expressed (integrative analysis) [file 13148_2024_1695_MOESM1_ESM.tif]
