# Supplementary material for: DNA methylation profile of inflammatory breast cancer and its impact on prognosis and outcome
Source: Clin Epigenetics. 2024 Jul 6;16:89. doi: 10.1186/s13148-024-01695-x (PMC11227707; doi:10.1186/s13148-024-01695-x)
Supplement: Supplementary file 3 — Table S2 Genetic variants found in TP53, PIK3CA, homologous recombination and mismatch repair genes among the 29 IBC cases assessed by t-NGS. [file 13148_2024_1695_MOESM3_ESM.docx]

**Table S2.** Genetic variants found in *TP53*, *PIK3CA*, homologous recombination and mismatch repair genes among the 28 IBC cases assessed by tNGS.

| **Sequenced cases** | ***TP53*** | ***PIK3CA*** | **homologous recombination genes** | **Mismatch repair genes** |
| --- | --- | --- | --- | --- |
| 1. IBC1 |  | P |  |  |
| 1. IBC2 |  | P |  |  |
| 1. IBC4 |  |  | *ATM* (VUS) |  |
| 1. IBC5 |  |  | *BRCA2* (P) |  |
| 1. IBC6 |  |  |  |  |
| 1. IBC7 |  |  | *BRCA2* (VUS_P) | *MLH1* (VUS) |
| 1. IBC8 | VUS |  |  |  |
| 1. IBC9 |  |  | *BRCA2* (P), *RAD51B* (VUS) and *POLD1* (VUS) |  |
| 1. IBC10 |  |  | *BRCA2* (P) |  |
| 1. IBC11 | P |  | *BRCA2* (P) and *RAD51B* (VUS) | *MSH6* (LP) |
| 1. IBC12 | VUS_P |  | *RAD51D* (VUS) | *PMS2* (VUS_P) |
| 1. IBC13 | LP |  | *BRCA2* (VUS) |  |
| 1. IBC14 | P |  | *BRCA2* (P) |  |
| 1. IBC15 |  |  | *PALB2* (P) | *MSH3* (VUS) |
| 1. IBC16 | P |  |  | *MSH3* (VUS) |
| 1. IBC17* | P |  | *ATM* (VUS) |  |
| 1. IBC18 | P | P | *BRCA2* (VUS) |  |
| 1. IBC20 | 4 (2 P and 2 LP) | P | *BRCA1* (P) | *MLH3* (VUS) |
| 1. IBC22 | P | P | *BRCA2, MUS81* (VUS) | *PMS2* (VUS) |
| 1. IBC25 |  |  |  |  |
| 1. IBC26 | P |  |  |  |
| 1. IBC66* | VUS |  | *ATM* (VUS) |  |
| 1. IBC67* | VUS |  | ATM (VUS) |  |
| 1. IBC68* | P | P |  |  |
| 1. IBC69* | VUS |  | ATM (VUS); MUS81(VUS) |  |
| 1. IBC70* | LP |  | ATM (VUS) |  |
| 1. IBC71* | P |  | RAD51B (P) |  |
| 1. IBC72* | VUS |  | BRCA1 (P) |  |

*Samples sequenced in the present study; the other samples were previously described in Faldoni et al, 2020.

MMR: mismatch repair; VUS: variant with uncertain significance, VUS_P: VUS potentially pathogenic, LP: likely pathogenic; P: pathogenic.
